# Sex and strain dependent differences in mucosal immunology and microbiota composition in mice

**DOI:** 10.1186/s13293-018-0186-6

**Published:** 2018-06-18

**Authors:** Marlies Elderman, Floor Hugenholtz, Clara Belzer, Mark Boekschoten, Adriaan van Beek, Bart de Haan, Huub Savelkoul, Paul de Vos, Marijke Faas

**Affiliations:** 1 Top Institute Food and Nutrition, Wageningen, the Netherlands; 2 Division of Medical Biology, Department of Pathology and Medical Biology, University of Groningen and University Medical Centre Groningen, 9713 GZ Groningen, the Netherlands; 3 Laboratory of Microbiology, Wageningen University and Research, 6703 WE Wageningen, the Netherlands; 4 Division of Human Nutrition, Wageningen University and Research, 6703 WE Wageningen, the Netherlands; 5 Cell Biology and Immunology, Wageningen University and Research, 6708 WD, Wageningen, the Netherlands; 6 Department of Obstetrics and Gynecology, University of Groningen and University Medical Centre Groningen, 9713 GZ Groningen, the Netherlands

**Keywords:** Microbiota, Sex differences, B6, BALB/c, Mesenteric lymph nodes, Colon, Intestinal immune populations, Mucosal immune populations

## Abstract

**Background:**

A dysbiosis in the intestinal microbiome plays a role in the pathogenesis of several immunological diseases. These diseases often show a sex bias, suggesting sex differences in immune responses and in the intestinal microbiome. We hypothesized that sex differences in immune responses are associated with sex differences in microbiota composition.

**Methods:**

Fecal microbiota composition (MITchip), mRNA expression in intestinal tissue (microarray), and immune cell populations in mesenteric lymph nodes (MLNs) were studied in male and female mice of two mouse strains (C57B1/6OlaHsd and Balb/cOlaHsd). Transcriptomics and microbiota data were combined to identify bacterial species which may potentially be related to sex-specific differences in intestinal immune related genes.

**Results:**

We found clear sex differences in intestinal microbiota species, diversity, and richness in healthy mice. However, the nature of the sex effects appeared to be determined by the mouse strain as different bacterial species were enriched in males and females of the two strains. For example, *Lactobacillus plantarum* and *Bacteroides distasonis* were enriched in B6 females as compared to B6 males, while *Bifidobacterium* was enriched BALB/c females as compared to BALB/c males. The strain-dependent sex effects were also observed in the expression of immunological genes in the colon. We found that the abundance of various bacteria (e.g., *Clostridium leptum et rel.*) which were enriched in B6 females positively correlated with the expression of several genes (e.g., *Il-2rb*, *Ccr3*, and *Cd80*) which could be related to immunological functions, such as inflammatory responses and migration of leukocytes. The abundance of several bacteria (e.g., *Faecalibacterium prausnitzii et rel.* and *Coprobacillus et rel.- Clostridium ramosum et rel.*) which were enriched in BALB/c males positively correlated to the expression of several genes (e.g., *Apoe*, *Il-1b*, and *Stat4*) related to several immunological functions, such as proliferation and quantity of lymphocytes. The net result was the same, since both mouse strains showed similar sex induced differences in immune cell populations in the MLNs.

**Conclusions:**

Our data suggests a correlation between microbiota and intestinal immune populations in a sex and strain-specific way. These findings may contribute to the development of more sex and genetic specific treatments for intestinal-related disorders.

**Electronic supplementary material:**

The online version of this article (10.1186/s13293-018-0186-6) contains supplementary material, which is available to authorized users.

## Background

The human gut harbors trillions of microbes [1]. The recent change of the traditional view that gut microbiota not only affect fermentation of food components, but also influence metabolism and immune status, has led to the realization that these microbes can impact health on different levels and that they are instrumental for maintaining health [2–4]. Microbes in the intestine can substantially be influenced by external factors such as diet and antibiotics, which may disturb the microbiota-host interactions in an undesirable way and can ultimately lead to disease [5]. However, these findings also demonstrate the potential to improve human health or to treat and prevent diseases by using nutrition or drugs [5, 6].

During recent years, a disbalance in intestinal microbiota communities (intestinal dysbiosis) has been found to play a significant role in the pathogenesis of a large number of immunological Western diseases, such as inflammatory bowel disease (IBD), other autoimmune diseases, and metabolic syndrome [7, 8]. This growing list of Western-world diseases correlates with changes in microbiota composition [9–11]. Microbiota-derived molecules, such as short-chain fatty acids (SCFAs), have been recognized to influence intestinal immune cells [12, 13]. For example, the SCFA butyrate has been shown to induce the differentiation of T regulatory (Tregs) in the colon [14]. Furthermore, some microbiota are involved in the generation of specific regulatory responses in T-cells [12, 15], while others stimulate specific T helper 17 (Th17) cell responses [16].

There is a sex bias in the prevalence of many of the aforementioned Western-world diseases [17–19]. It is currently unknown whether this sex bias is influenced by sex-dependent differences in immune modulating microbiota. Sex differences in peripheral immune responses are well known [20, 21], and in general, it is thought that females have a stronger innate and adaptive immune response as compared with males [22]. However, also here the influence of microbiota differences has gained minor attention. Although several studies showed the existence of sex differences in microbiota composition [23–28], minor knowledge is available on sex differences in intestinal immunology and on the influence of microbiota on sex-specific immune responses. Markle et al. and Yurkovetskiy et al. both showed that microbiota and sex hormones contribute to the effector mechanism of sex bias in type 1 diabetes in non-obese diabetic (NOD) mice [23, 27]. If sex-dependent microbiota differences underlie the differences in sex-specific immunity, it might open new venues for designing effective strategies to improve human health by manipulating microbiota and associated immune responses in a sex-specific way. Therefore, in this study, we investigated the relationship between sex differences in immune populations and sex differences in microbiota in healthy mice.

As various factors, such as the reproductive condition, genetic background, and diet, can interfere with the sex effects [25, 28, 29], we compared male and female mice from two different strains (C57B1/6OlaHsd (B6) and Balb/cOlaHsd (BALB/c)) with two difference genetic backgrounds under exactly the same reproductive and dietary conditions. The B6 and BALB/c strains were specifically chosen, because of their known difference in intestinal immune responses during Dextran sulfate sodium (DSS)-induced colitis [30, 31]. In mice from both sexes and mouse strains, we analyzed the microbiota composition and we performed a microarray on colonic tissue. We combined the transcriptomics data and the microbiota data and performed a bio-mathematical analysis, in order to find bacterial species, which may potentially be related to sex-specific differences in intestinal gene expression. Subsequently, key immunological changes found in the microarray were studied in the MLN using flow cytometry. The MLN are used as intestinal reference site, as this is the place where lymphocytes are primed and activated by intestinal DCs deriving from the gut [32].

## Methods

### Study design

This study was designed to assess the effect of sex on intestinal microbiota and intestinal immune cell composition in mice. Two different mice strains were used; C57B1/6OlaHsd (B6) and Balb/cOlaHsd (BALB/c). In both strains (*n* = 20 per strain) two groups were present; female and male mice (*n* = 10 per sex). Between an age of 11 and 23 weeks, all mice were sacrificed by cervical dislocation under anesthesia (isoflurane and oxygen). Table 1 provides an overview of the characteristics of all mice (*n* = 10 per group), and Table 2 provides an overview of the characteristics of the mice which were, per strain and sex, randomly selected from two cages for microbiota and microarray analysis (*n* = 5 per group). Subsequently, their mesenteric lymph nodes (MLN) were removed for immune cell analysis. During sacrifice, feces from the distal colon were collected for MITChip analysis. Approximately 1 cm of proximal colon was removed for microarray analysis. All female mice were sacrificed during the diestrus phase of their ovarian cycle to ensure low stable levels progesterone and estrogens.

**Table 1:** Overview of mice characteristics. No significant differences in age at sacrifice were found between males and females within each mouse strain (Kruskal-Wallis test followed by Dunn’s multiple comparison test, *p* < 0.05)

	BALB/c male	BALB/c female	B6 male	B6 female
Number of mice	10	10	10	10
Age at sacrifice (weeks)	12.8 (2.4)	15.3 (3.4)	17.0 (3.4)	18.8 (3.3)

**Table 2:** Overview of mice characteristics selected for microbiota and microarray analysis. No significant differences in age at sacrifice were found between males and females within each mouse strain (Kruskal-Wallis test followed by Dunn’s multiple comparison test, *p* < 0.05)

	BALB/c male	BALB/c female	B6 male	B6 female
Number of mice	5	5	5	5
Age at sacrifice (weeks)	13.9 (4.3)	18.5 (3.5)	19.7 (1.4)	18.2 (0.4)

### Mice

Male and female wild-type B6 and BALB/c mice were purchased from Harlan (Harlan, Horst, the Netherlands) at an age of 8 weeks. Mice were co-housed (five mice per cage, according to sex and strain) in isolated ventilated cages to limit environmental influences. The animals had ad libitum access to food (D12450B diet from Research Diets Services, Wijk bij Duurstede, the Netherlands) and water.

### Bacterial DNA extraction and microbiota profiling

Total DNA was extracted from the fecal samples (*n* = 5 mice per group, divided over at least two different cages) using the repeated bead-beating-plus column (RBB + C) method [33]. The microbiota composition was determined using the mouse intestinal tract chip (MITChip), a diagnostic 16S rRNA array, which consists of 3580 unique probes designed to profile murine intestinal microbiota [34]. Briefly, for MITChip, 16S rRNA gene amplification of the bacterial DNA, in vitro transcription, labeling, and hybridization were carried out as described previously [35]. Data were normalized and analyzed using a set of R-based scripts in combination with a custom-designed relational database, which operates under the MySQL database management system. For microbial profiling, the Robust Probabilistic Averaging (RPA) signal intensities of 2667 specific probes for the 94 genus-level bacterial groups detected on the MITChip, were used [36]. Diversity calculations were performed using a microbiome R-script package (https://github.com/microbiome). The redundancy analysis (RDA) was performed in Canoco 5.0, where variables were tested for their significance by the Monte Carlo permutation and visualized in triplots [37].

### Intestinal microarray analysis

For microarray analysis, RNA was purified from the proximal colon of mice (*n* = 5 per group) using TRIzol (Life Technologies, Calsbad, CA, USA) followed by an additional round of purification with RNeasy Minikit columns (Qiagen, Venlo, the Netherlands). The quality of RNA was determined using RNA 6000 nanochips on the Agilent 2100 bioanalyzer (Agilent Technologies, Amsterdam, the Netherlands). Purified RNA (100 ng) was labeled with the Affymetrix WT PLUS reagent kit (Affymetrix, Santa Clara, CA, USA) and hybridized to an Affymetrix Mouse Gene 1.1 ST array plate (Affymetrix, Santa Clara, CA, USA). Hybridization, washing, and scanning were carried out on an Affymetrix GeneTitan platform according to the manufacturer’s instructions. Arrays were normalized using the robust multiarray average method [38, 39]. Probe sets were defined according to Dai et al. (2005) [40]. In this method, probes are assigned to Entrez IDs as a unique gene identifier. The *p* values were calculated using an intensity-based moderated *t* statistic (IBMT) [41]. Only probe sets with a fold-change of at least 1.2 (up/down) and a *p* value < 0.05 were considered to be significantly different. The microarray data was validated by real-time quantitative PCR (see Additional file 1 for the used method and results).

To gain insight into the biological role of the sexually dimorphically expressed genes, we investigated the functions in which these genes are involved using Ingenuity Pathway Analysis (IPA) (Ingenuity System). The IPA output includes biological functions and signaling pathways with statistical assessment of the significance of their representation based on Fisher’s exact test. Here, this test calculates the probability that genes participate in a given biological function relative to their occurrence in all other biological function annotations. Our IPA analyses included comparison of differentially regulated genes in the colon of males and females in both B6 and BALB/c mice.

### Multivariate integration and correlation analysis

To gain insight in the relationship between the colonic gene expression and microbiota composition, the microarray and MITChip datasets were combined, using the linear multivariate method partial least squares (PLS) [42], as described previously [43]. This integration of datasets per individual mouse gives a direct correlation between gene expression and microbiota composition in these samples. For 15 mice, both gene expression and data on microbiota composition were available (*n* = 3–5 per group). Both datasets were log2 transformed before analysis, and the canonical correlation framework of PLS was used [44]. The correlation matrices were visualized in clustered image maps [45]. Analyses were performed in R using the library mixOmics [46]. A positive correlation between bacteria and genes indicates that a higher abundance of the bacteria is associated with a higher expression of the particular cluster of genes. A negative correlation between bacteria and genes indicates that a lower abundance of the bacteria is associated with a lower expression of the particular cluster of genes.

### Mesenteric lymph node cell isolation

Single cell suspensions of the MLN were made by mechanical disruption of the tissues between two object glasses in 2 ml ice cold RPMI containing 10% heat inactivated fetal calf serum (FCS). Falcon tubes with cell strainer caps (Corning, Amsterdam, the Netherlands) (35 μm) were used to remove cell clumps before the cells were counted and used for staining.

### Cell staining

MLN cells were stained for T lymphocytes (CD3^+^), T cytotoxic cells (CD8^+^) and T helper cells (CD4^+^). Expression of CD69, α4ß7, CD62L, and CD44 was measured within the CD8^+^ and CD4^+^ cell subsets. Specifications of the antibodies used are described in Table 3. All antibodies were diluted in a volume of 25 μl, supplemented to a volume of 25 μl with FACS buffer (PBS + 10% FCS (*v/v*)). Approximately 0.5 × 10^6^ MLN cells were incubated for 20 min in FACS buffer (10% FCS (*v/v*)) containing 20% (*v/v*) normal rat serum (Jackson, Newmarket, UK) and 2% (*v/v*) Fc block (CD16/32) (Biolegend, Uithoorn, the Netherlands) to prevent non-specific antibody binding followed by incubation in the primary antibody mix for 30 min. Next, the cells were incubated with a biotinylated antibody (streptavidin-Pacific Orange) for 30 min and subsequently fixed in FACS lysing solution (BD Biosciences, Breda, the Netherlands) for 30 min. Washing was performed in between all incubation steps. The whole procedure was performed on ice and in the dark. Isotype control antibodies were used at the same concentration and purchased from the same company as the primary and secondary antibodies.

**Table 3:** Antibody specifications

Specificity	Clone name	Fluorchrome	Concentration	Dilution^a^	Supplier
CD3	17A2	Pacific blue	0.5 mg/ml	80×	Biolegend
CD8	53–6.7	A700	0.5 mg/ml	50×	Biolegend
CD4	GK1.5	PE-Cy7	0.2 mg/ml	100×	Biolegend
CD69	H1.2F3	FitC	0.5 mg/ml	25×	Biolegend
α4β7	DATK32	APC	0.2 mg/ml	25×	Biolegend
CD62L	MEL-14	Biotin	0.5 mg/ml	200×	Biolegend
		Streptavidin-Pacific Orange	1 mg/ml	100×	ThermoFisher
CD44	IM7	APC-Cy7	0.2 mg/ml	100×	Biolegend

^a^Dilution used in a total volume of 25 μl supplemented with PBS + 10% FCS

### Flow cytometry

Cell samples were analyzed using the LSR-II Flow Cytometer system (BD Biosciences, Breda, the Netherlands) using FACS Diva software. Analysis was performed using FlowJo version 10 software (FlowJo, LLC, OR, USA). Lymphocytes were gated based on size in the forward side scatter plot, and T cells were determined by selecting CD3^+^ cells. Within the CD3^+^ cells, CD4^+^ and CD8^+^ cells were selected. Within both the CD4^+^ and CD8^+^ population, the percentage of cells expressing CD69, α4ß7, CD62L, and CD44 was measured. Therefore, all their isotype controls were set at 1% and these gates were copied to the samples with the antibody mix (see also Figs. 4 and 5).

### Statistical analysis

For flow cytometry data, Shannon diversity, microbiota richness and the Firmicutes/Bacteroidetes ratio, data are expressed as the mean with standard error of the mean (SEM). The Kolmogorov-Smirnov test was used to determine normal distribution of the data. When the data were not normally distributed a log transformation was performed before analysis. The data were analyzed with a two-way ANOVA followed by a Bonferroni post-test when interaction was found. For analyzing significant effects of sex and strain on the abundance of bacteria groups a Mann–Whitney *U* test was used. *p* values of 0.05 or smaller were considered statistically significant and *p* values between 0.05 and 0.1 were defined as a trend.

## Results

### Sex influenced intestinal microbiota composition in a mouse strain-dependent way

**Fig. 1:**
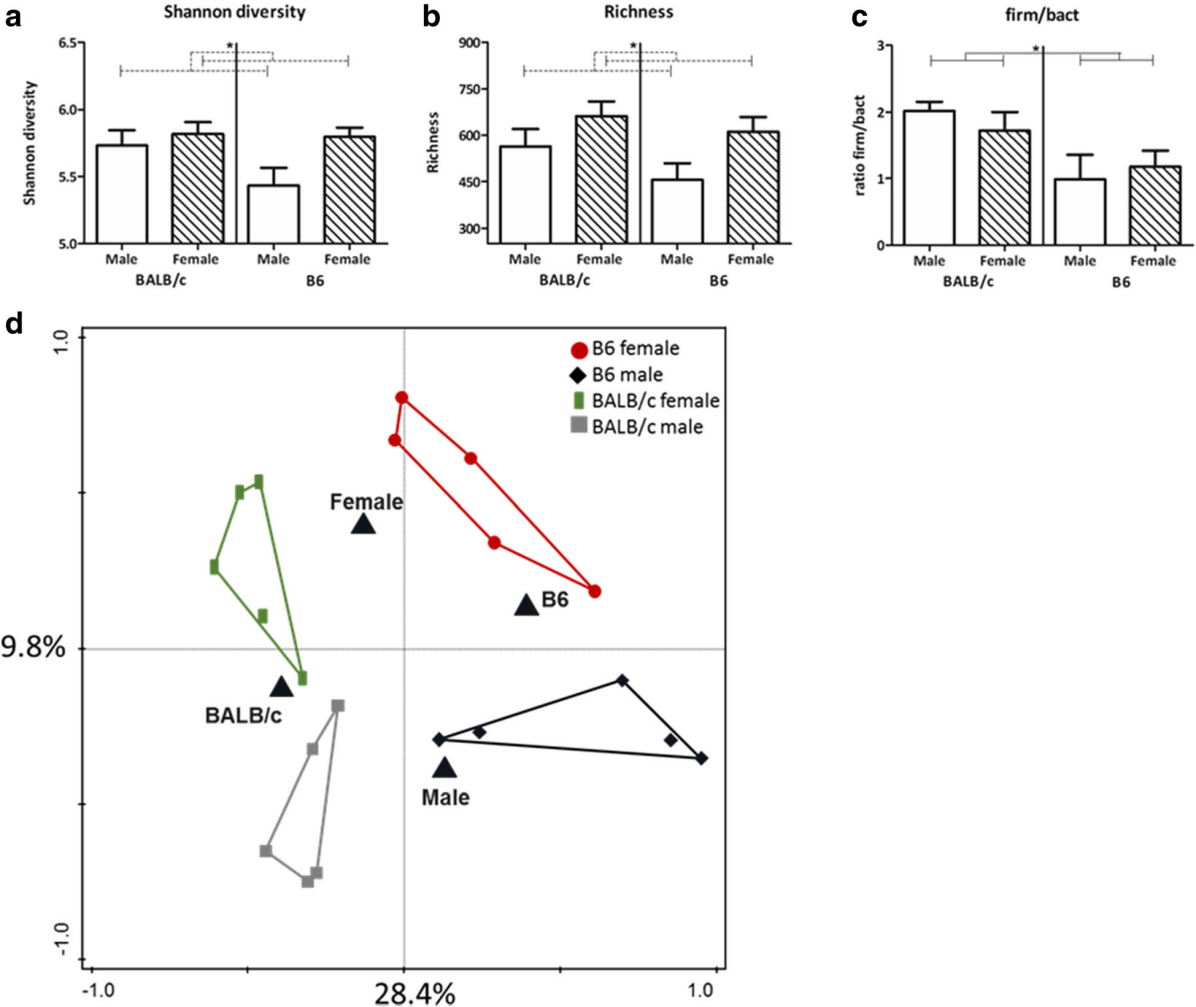
Effect of sex and strain on fecal microbiota characteristics. Shannon diversity (**a**), richness (**b**), and the Firmicutes/Bacteroidetes (**c**) ratio in the fecal microbiota of male and female BALB/c and B6 mice (5 mice per group). Results are shown as mean + SEM and were tested for overall strain and sex effects using a two-way ANOVA followed by a Bonferroni post hoc test to test for strain-specific sex effects when interaction was found. Significant strain effects are indicated with solid lines and significant sex effects are indicated with dashed lines (*p* < 0.05). RDA plot showing the variation explained by the components genotype and sex (five mice per group) (**d**). The total variation that can be explained by the variables genotype (26.5%) and sex (11.6%) is 38.1%. Both variables are significant in explaining the variation (Monte Carlo permutation, *p* < 0.05)

The microbial composition in the feces of males and females of both BALB/c and B6 mice was determined using the phylogenetic microarray, the mouse intestinal tract Chip (MITChip). Additionally, we determined the richness (number of unique species) and Shannon diversity (calculation between richness and evenness (abundances over species) of the microbiota composition. Overall, males had a lower diversity (two-way ANOVA, *p* = 0.046) and richness (two-way ANOVA, *p* = 0.027) than females (Fig. 1a, b), while there was no effect of strain. A higher ratio of Firmicutes/Bacteroidetes was found in BALB/c mice (two-way ANOVA, *p* = 0.009); however, this ratio was not influenced by sex (Fig. 1c).

Redundancy analysis showed that the total variation in microbiota composition explained by the variables genotype and sex is 38.1%. Sex explained 11.6% of the variance in microbiota composition (Fig. 1d). This was, however, mouse strain dependent, since the variable strain explained 26.5% of the variance in the microbial composition. Table 4 provides an overview of the relative abundance of the bacteria groups and the differences in relative abundance between males and females within each mouse strain.

**Table 4:** Relative abundance of bacteria groups in BALB/c and B6 male and female mice (*n* = 5). Differences between males and females within each mouse strain were determined with a Mann–Whitney *U* test. Significant differences are highlighted in italics

Bacteria group	BALB/c female	BALB/c male	*p* value	B6 female	B6 male	*p* value B6
*Acholeplasma et rel.*	0.0041%	0.0061%	0.095	0.0084%	0.0096%	0.690
*Aerococcus urinaeequi et rel.*	0.0074%	0.0117%	0.095	0.0098%	0.0097%	0.841
*Alistipes et rel.*	0.9156%	0.3753%	0.095	0.5414%	0.3036%	0.095
*Allobaculum et rel.*	0.2716%	0.2299%	0.548	4.0636%	7.4252%	0.095
*Anaerovorax et rel.*	0.0644%	0.0841%	0.222	0.0513%	0.0578%	0.548
*Atopobium*	0.0046%	0.0078%	*0.032*	0.0096%	0.0150%	0.310
*Bacteroides distasonis et rel.*	0.1100%	0.0266%	0.690	0.1126%	0.0373%	*0.016*
*Bacteroides vulgatus et rel.*	0.2397%	0.0924%	0.151	0.0631%	0.0585%	0.548
*Bifidobacterium*	0.0828%	0.0250%	*0.008*	1.8595%	2.9444%	1.000
*Bilophila et rel.*	0.0179%	0.0196%	0.421	0.0155%	0.0138%	1.000
*Catenibacterium*	0.0140%	0.0202%	0.056	0.0259%	0.0330%	0.548
*Clostridium difficile et rel.*	0.9357%	0.7979%	0.310	1.1169%	0.2468%	*0.032*
*Clostridium herbivorans et rel.*	0.0083%	0.0134%	0.095	0.0132%	0.0143%	0.690
*Clostridium lactifermentans et rel.*	0.3127%	0.3251%	0.841	0.1212%	0.1025%	0.421
*Clostridium leptum et rel.*	0.5591%	0.4151%	0.095	0.4190%	0.1997%	*0.008*
*Clostridium perfringens et rel.*	2.3301%	2.7132%	0.690	0.3328%	0.0963%	*0.016*
*Clostridium symbosium et rel.*	0.5136%	0.5040%	0.841	0.7587%	0.3233%	0.056
*Coprobacillus catenoformis et rel.*	0.0230%	0.0483%	*0.008*	0.0273%	0.0410%	0.222
*Coprobacillus et rel.- Clostridium ramosum et rel.*	0.1377%	0.2504%	*0.032*	0.2859%	0.3510%	0.690
*Corynebacterium et rel.*	0.0067%	0.0104%	0.056	0.0108%	0.0128%	0.548
*Desulfovibrio et rel.*	0.0789%	0.0904%	0.548	0.0454%	0.0355%	0.841
*Dialister et rel.*	0.0027%	0.0044%	0.095	0.0044%	0.0048%	0.548
*Dorea et rel.*	3.4135%	8.6217%	0.095	2.3603%	2.4100%	0.841
*Eggerthella et rel.*	0.2295%	0.4959%	*0.016*	0.2927%	0.5021%	*0.032*
*Enterococcus*	0.2846%	0.4653%	0.310	0.1058%	0.0130%	*0.008*
*Eubacterium cylindroides et rel.*	0.0141%	0.0225%	0.095	0.0232%	0.0263%	0.690
*Eubacterium hallii et rel.*	0.0026%	0.0043%	0.095	0.0043%	0.0047%	0.548
*Eubacterium siraeum et rel.*	0.0084%	0.0139%	0.095	0.0135%	0.0145%	0.548
*Faecalibacterium prausnitzii et rel.*	0.0029%	0.0046%	0.095	0.0051%	0.0050%	0.690
*Lachnobacillus bovis et rel.*	1.9742%	2.1309%	0.841	1.6788%	1.0041%	0.421
*Lachnospira pectinoschiza et rel.*	0.9649%	0.4554%	0.310	0.3867%	0.3047%	0.421
*Lactobacillus acidophilus et rel.*	0.0418%	0.0592%	0.222	0.4314%	0.0518%	0.016
*Lactobacillus delbrueckii et rel.*	0.0062%	0.0097%	0.222	0.0176%	0.0100%	0.548
*Lactobacillus paracasei et rel.*	0.0121%	0.0182%	0.095	0.0153%	0.0129%	0.690
*Lactobacillus plantarum et rel.*	0.0222%	0.0300%	0.222	0.3977%	0.0452%	*0.032*
*Lactobacillus salivarius et rel.*	0.5801%	1.9226%	0.095	1.1330%	0.7385%	1.000
*Lactococcus et rel.*	0.0029%	0.0047%	0.056	0.0048%	0.0054%	0.548
*Mucispirillum schaedleri et rel.*	0.1270%	0.1483%	0.690	0.0544%	0.0260%	0.222
*Papillibacter cinnamivorans et rel.*	0.1624%	0.1898%	0.548	0.0784%	0.0734%	0.841
*Propionibacterium*	0.0036%	0.0055%	0.056	0.0067%	0.0082%	0.548
*Roseburia intestinalis et rel.*	0.0210%	0.0344%	0.056	0.0324%	0.0333%	0.690
*Ruminobacter amylophilus et rel.*	0.0035%	0.0056%	0.056	0.0054%	0.0056%	0.548
*Ruminococcus callidus et rel*	0.0197%	0.0268%	0.095	0.0241%	0.0242%	0.548
*Ruminococcus obeum et rel.*	0.0063%	0.0107%	0.095	0.0091%	0.0098%	0.690
*Solobacterium moorei et rel.*	0.0116%	0.0184%	0.056	0.0231%	0.0275%	0.690
*Sporobacter termitidis et rel.*	17.1016%	13.7342%	0.421	8.8797%	5.9207%	0.222
*Staphylococcus aureus et rel.*	0.0171%	0.0669%	*0.008*	0.0216%	0.0238%	0.690
*Streptococcus intermedius et rel.*	0.0067%	0.0112%	*0.032*	0.0101%	0.0085%	0.690
*Subdoligranulum et rel.*	0.0046%	0.0073%	0.056	0.0061%	0.0065%	0.548
*Sutterella wadsworthia et rel.*	0.0127%	0.0205%	0.056	0.0300%	0.1068%	0.310
*Turicibacter et rel.*	0.6261%	0.4110%	0.421	0.3876%	0.0261%	*0.008*
*Unclassified Bacteroidetes*	0.0029%	0.0044%	0.095	0.0046%	0.0049%	0.548
*Unclassified Clostridiales I*	0.0617%	0.1277%	0.056	0.1199%	0.1494%	0.690
*Unclassified Clostridiales II*	0.1700%	0.2517%	*0.032*	0.1490%	0.1350%	1.000
*Unclassified Clostridiales XIVa–close to Anaerostipes caccae*	0.0210%	0.0329%	0.056	0.0305%	0.0311%	0.690
*Unclassified Clostridiales XVI*	0.0751%	0.1497%	0.056	0.1577%	0.1977%	0.548
*Unclassified Mollicutes*	0.1218%	0.1663%	*0.016*	0.2794%	0.3999%	0.548
*Unclassified Porphyromonadaceae*	34.4807%	31.7121%	0.841	44.8531%	52.5080%	0.421
*Unclassified Prevotella*	1.1225%	0.3418%	0.690	0.2821%	0.2037%	0.095
*Uncultured Clostridiales*	0.2248%	0.1868%	0.151	0.1670%	0.1381%	0.690
*Veilonella*	0.0026%	0.0043%	0.095	0.0044%	0.0048%	0.548

### Sex influenced gene expression profiles in the colon in a mouse strain-dependent way

Next, we performed a microarray analysis on the colon of male and female mice. We performed this analysis in both BALB/c and B6 mice to identify potential mouse strain-dependent sex effects. In the colon, a total of 1110 genes in BALB/c mice and 3309 genes in B6 mice were differently expressed between males and females. The two mice strains shared 531 genes that were differently expressed between males and females in the colon (both up- and downregulated).

To gain insight into the biological role of the genes which were differently expressed between the sexes, we first studied the physiological activities and molecular and cellular functions per mouse strain in which these genes are involved using Ingenuity Pathway Analysis (IPA). We specifically focused on functions related to immunology. In the colon in both mouse strains, IPA showed enrichment for genes related to, among others, hematological system development and immune cell trafficking (Table 5).

**Table 5:** The top physiological activities and molecular and cellular functions related to the sexually dimorphic expressed genes in the proximal colon of both BALB/c and B6 mice (fold-change of 1.2 (up/down) and a *p* value < 0.05)

Group	Physiological system development and function	*p* value	# Mol.^a^
BALB/c	Hematological system development and function	6.67E-04-1.74E-13	170
	Tissue morphology	6.56E-04-4.43E-13	157
	Cell-mediated immune response	6.37E-04-3.59E-12	67
	Immune cell trafficking	6.67E-04-3.59E-12	110
*B6*	Hematopoiesis	6.44E-04-2.71E-09	80
	Tissue morphology	2.67E-07-1.21E-29	614
	Immune cell trafficking	4.90E-07-3.62E-29	360
	Hematological system development and function	4.90E-07-3.07E-26	577
	Cardiovascular system development and function	2.56E-07-2.40E-24	424
	Organismal development	4.33E-07-2.40E-24	808

^a^Number of molecules included in the indicated functions

Subsequently, we analyzed in more detail the functions of the genes that were differently expressed between the sexes and in both mouse strains. IPA listed 500 functions that were enriched, in both or one of the two strains, from the genes displaying sexually dimorphic expression. Again, we specifically focused on functions related to immune response (Table 6). We found that sex altered the expression of genes related to several immune functions in the colon in both mouse strains. Many functions were related to T lymphocytes, and more specifically to T cell activation, development, proliferation capacity, and homing and migration. However, for most functions, we found that sex did not have the same effect in both strains. For example, the quantity of T lymphocytes was increased in BALB/c males as compared with BALB/c females, while reduced in B6 males as compared with B6 females.

**Table 6:** Selection of immunological functions that are related to the genes with a different expression in males and females in both BALB/c and B6 mice in the proximal colon. The *z* score gives an indication of the activation or inhibition of the functions in males versus females. The number of molecules includes the number of molecules involved in the indicated function (fold-change of 1.2 (up/down) and a *p* value < 0.05)

Diseases or functions annotation	*z* score BALB/c	z score B6	*p* value BALB/c	*p* value B6	# Mol. BALB/c^a^	# Mol. B6^a^
Quantity of leukocytes	2.743	− 5.066	2.57E-11	4.64E-21	113	292
Quantity of lymphocytes	2.803	− 5.853	2.71E-09	2.45E-14	87	215
Quantity of T lymphocytes	2.638	−4.628	4.40E-07	1.46E-09	64	154
Quantity of granulocytes	− 0.018	0.063	5.13E-07	4.60E-12	42	104
Quantity of antigen presenting cells	2.556	− 1.933	6.91E-06	3.42E-08	35	82
Quantity of macrophages	1.925		1.03E-04		25	
Quantity of B lymphocytes	1.750	− 3.840	3.85E-05	3.11E-09	40	104
Quantity of dendritic cells	2.980		1.62E-04		17	
Proliferation of immune cells	0.895	− 2.700	5.17E-11	3.03E-12	100	228
Proliferation of lymphocytes	1.046	− 1.941	5.22E-10	3.57E-11	91	208
Proliferation of T lymphocytes	0.311		9.85E-10		78	
Homing of leukocytes	4.371	− 4.686	1.81E-08	2.24E-12	52	122
Homing of lymphocytes	4.226		2.67E-08		28	
Homing of T lymphocytes	3.798		4.00E-10		26	
Homing of helper T lymphocytes	2.805		1.41E-06		9	
Homing of regulatory T lymphocytes	1.980		1.69E-04		4	
Activation of leukocytes	2.053	− 3.085	1.15E-05	4.66E-18	74	225
Activation of T lymphocytes	1.303	− 1.944	1.01E-04	1.88E-08	40	105
Differentiation of neutrophils	0.599		2.62E-04		8	
Differentiation of leukocytes	2.423	− 3.410	6.65E-04	2.40E-10	64	190
Differentiation of T lymphocytes	0.697		6.75E-04		39	
T cell development	1.636		3.74E-04		51	
Inflammatory response	3.496	− 3.851	7.30E-10	1.19E-22	95	259
Bacterial Infections	0.179		2.86E-05		46	

^a^Number of molecules included in the indicated functions

### Correlation between microbiota composition and gene expression profile in the colon

**Fig. 2:**
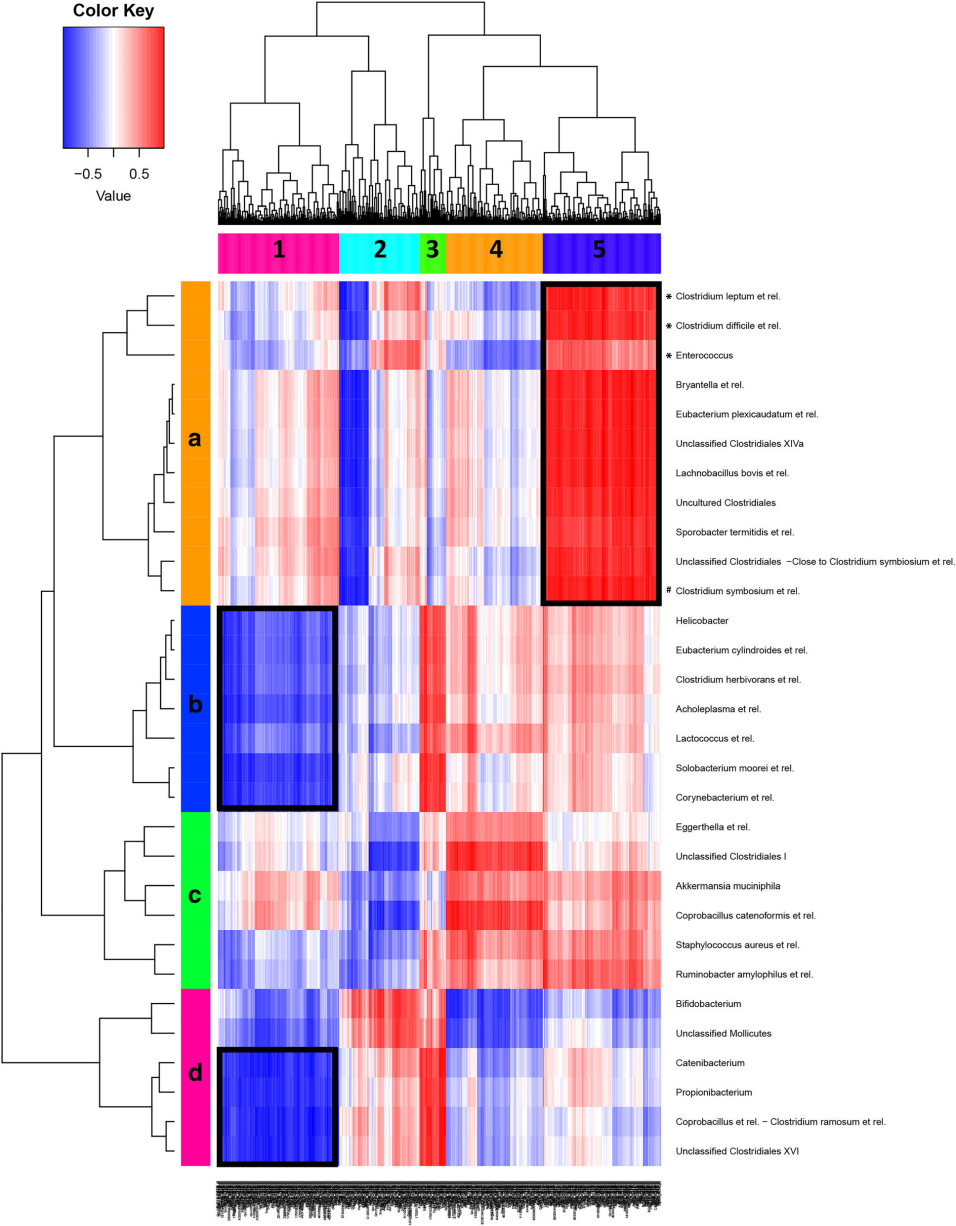
Correlation between microbiota species and gene expression in the colon of B6 mice. Heatmap of correlation analysis of MITChip (vertical) and microarray (horizontal) datasets of male and female B6 mice. The integration of datasets was done per individual mouse (five mice per group) and gives the direct correlations between gene expression and microbiota composition from these samples. In deep red, the cluster of genes that most positively correlated with a respective group of bacteria. In deep blue, the cluster of genes that most negatively correlated with a respective group of bacteria. Five main gene clusters (1–5) and four main bacteria clusters (A-D) were identified. The cluster framed in black is discussed in more detail in the text. A positive correlation between bacteria and genes indicates that a higher abundance of the bacteria is associated with a higher expression of the particular cluster of genes. A negative correlation between bacteria and genes indicates that a lower abundance of the bacteria is associated with a lower expression of the particular cluster of genes. The functions to which these genes are related to are presented in Table 7. Note that the bacteria and intestinal genes that were selected for the correlation were the ones most explanatory for variation between sex and mouse strain, and therefore the genes in a specific cluster do not necessarily have a significantly different expression between the sexes. Moreover, a positive correlation between bacteria and genes not necessary indicates that the particular function related to these genes is upregulated, as the genes involved may also have a suppressive effect on the function. Asterisks (*) and hashtags (#) indicate that the specific bacteria has a significantly/or tend to have a higher abundance in B6 females as compared to B6 males, respectively

**Fig. 3:**
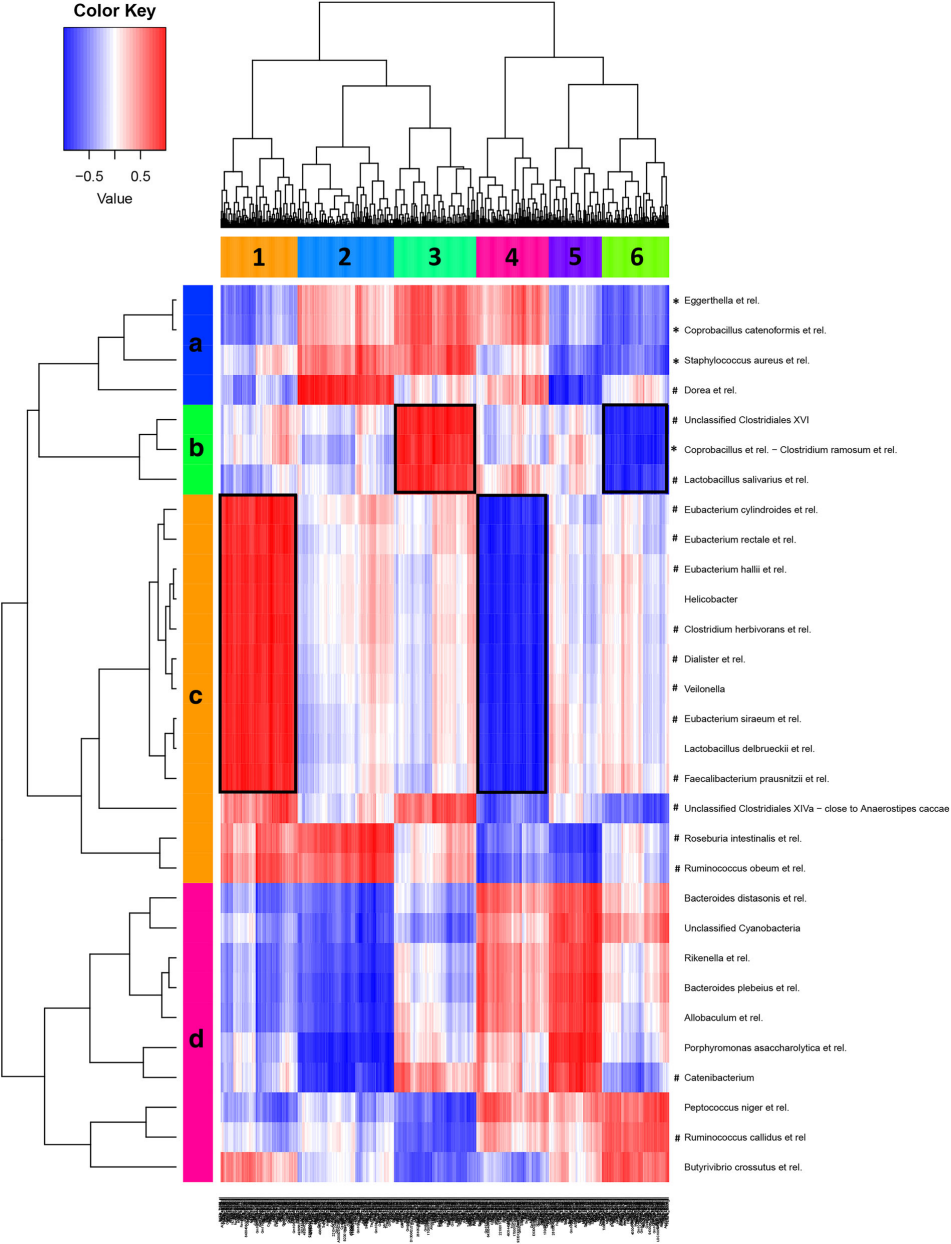
Correlation between microbiota species and gene expression in the colon of BALB/c mice. Heatmap of correlation analysis of MITChip (vertical) and microarray (horizontal) datasets of male and female BALB/c mice. The integration of datasets was done per individual mouse (five mice per group) and gives the direct correlations between gene expression and microbiota composition from these samples. In deep red, the cluster of genes that most positively correlated with a respective group of bacteria. In deep blue, the cluster of genes that most negatively correlated with a respective group of bacteria. Six main gene clusters (1–6) and four main bacteria clusters (A–D) were identified. The clusters framed in black are discussed in more detail in the text. A positive correlation between bacteria and genes indicates that a higher abundance of the bacteria is associated with a higher expression of the particular cluster of genes. A negative correlation between bacteria and genes indicates that a lower abundance of the bacteria is associated with a lower expression of the particular cluster of genes. The functions to which these genes are related to are presented in Table 7. Note that the bacteria and intestinal genes that were selected for the correlation were the ones most explanatory for variation between sex and mouse strain, and therefore the genes in a specific cluster do not necessarily have a significantly different expression between the sexes. Moreover, a positive correlation between bacteria and genes not necessary indicates that the particular function related to these genes is upregulated, as the genes involved may also have a suppressive effect on the function. Asterisks (*) and hashtags (#) indicate that the specific bacteria has a significantly/or tend to have a higher abundance in BALB/c males as compared to BALB/c females, respectively

To investigate the relation between microbiota species and immunological gene expression, we combined microbiota and colonic gene expression data from each BALB/c and B6 male and female mice individually, to evaluate direct correlations between gene expression and microbiota composition in these samples. We performed two separate correlations within each mouse strain to determine the effect of sex independent of mouse strain (Figs. 2 and 3). We integrated these datasets using a PLS-based canonical correlation approach. In total, 600 genes and 30 bacterial groups were retained for the first three components, and clustering of the correlation coefficients revealed six main clusters of host genes that correlated positively (red) or negatively (blue) to specific bacteria.

Within the B6 strain (Fig. 2), a strong positive correlation was found between gene expression cluster 5 and several bacteria which were or tended to be enriched in the females (such as Clostridium *leptum et rel.*, *Clostridium difficile et rel.*, *Enterococcus*, and *Clostridium symbosium et rel.*). The genes in this cluster are related to, among others, inflammatory response and migration of leukocytes (Table 7). Within the BALB/c strain (Fig. 3), we found four gene expression clusters which strongly correlated to certain bacteria groups. Both gene expression clusters 1 and 3 showed a strong positive correlation with bacteria which tended to be enriched in males (*Eubacterium cylindroides et rel*., *Eubacterium hallii et rel*., *Clostridium herbivorans et rel. Dialister et rel*., *Veilonella*, *Eubacterium siraeum et rel.*, and *Faecalibacterium prausnitzii et rel.* in cluster 1 and *Unclassified Clostridiales XVI*, *Coprobacillus et rel.-Clostridium ramosum et rel.*, and *Lactobacillus salivarius et rel.* in cluster 3). The genes in cluster 1 are related to, among others, proliferation of lymphocytes and quantity of leukocytes, whereas the genes in cluster 3 are related to, among others, expansion of T (helper) lymphocytes (Table 7). The gene expression clusters 4 and 6 showed a strong negative correlation with bacteria which tended to be enriched in males. Gene cluster 4 is related to, among others, quantity of leukocytes and chemotaxis of phagocytes, whereas gene cluster 6 is not related to immune functions (Table 7). The bacteria and intestinal genes that were selected for the correlation were the ones most explanatory for variation between sex and mouse strain, and therefore the genes in a specific cluster do not necessarily have a significantly different expression between the sexes. Moreover, a positive correlation between bacteria and genes not necessarily indicates that the particular function related to these genes is upregulated, as the genes involved may also have a suppressive effect on the function.

**Table 7:** Selection of immunological functions that are related to the genes in cluster 5 (B6 mice) and clusters 1, 3, and 6 (BALB/c mice) from the correlation analysis of MITChip and microarray

Gene cluster	Diseases or Functions Annotation	*p* value	# Mol.
Cluster 5 (B6)	Inflammatory response	6.46E-05	20
	Leukocyte migration	3.55E-04	20
	Infection of CD4+ T-lymphocytes	4.81E-04	2
	Quantity of leukocytes	6.13E-04	20
	Activation of leukocytes	8.90E-04	16
Cluster 1 (BALB/c)	Proliferation of lymphocytes	5.22E-05	34
	Quantity of leukocytes	5.78E-05	40
	Quantity of lymphoid cells	7.54E-05	33
	Quantity of myeloid cells	1.24E-04	24
	Quantity of mononuclear leukocytes	1.53E-04	33
Cluster 3 (BALB/c)	Expansion of T lymphocytes	1.60E-05	7
	Expansion of helper T lymphocytes	2.12E-05	4
	Immune response of leukocytes	1.06E-04	9
	Immune response of phagocytes	1.64E-04	7
	Leukocyte migration	5.60E-04	15
Cluster 4 (BALB/c)	Cell death of chronic lymphocytic leukemia B cells	1.65E-05	3
	Quantity of leukocytes	2.83E-05	17
	Chemotaxis of phagocytes	3.32E-05	9
	Quantity of mononuclear leukocytes	1.17E-04	14
	Quantity of phagocytes	1.32E-04	10

^a^Number of molecules included in the indicated functions

### Both sex and strain influenced T cell activation, migration, and maturation in the mesenteric lymph nodes

**Fig. 4:**
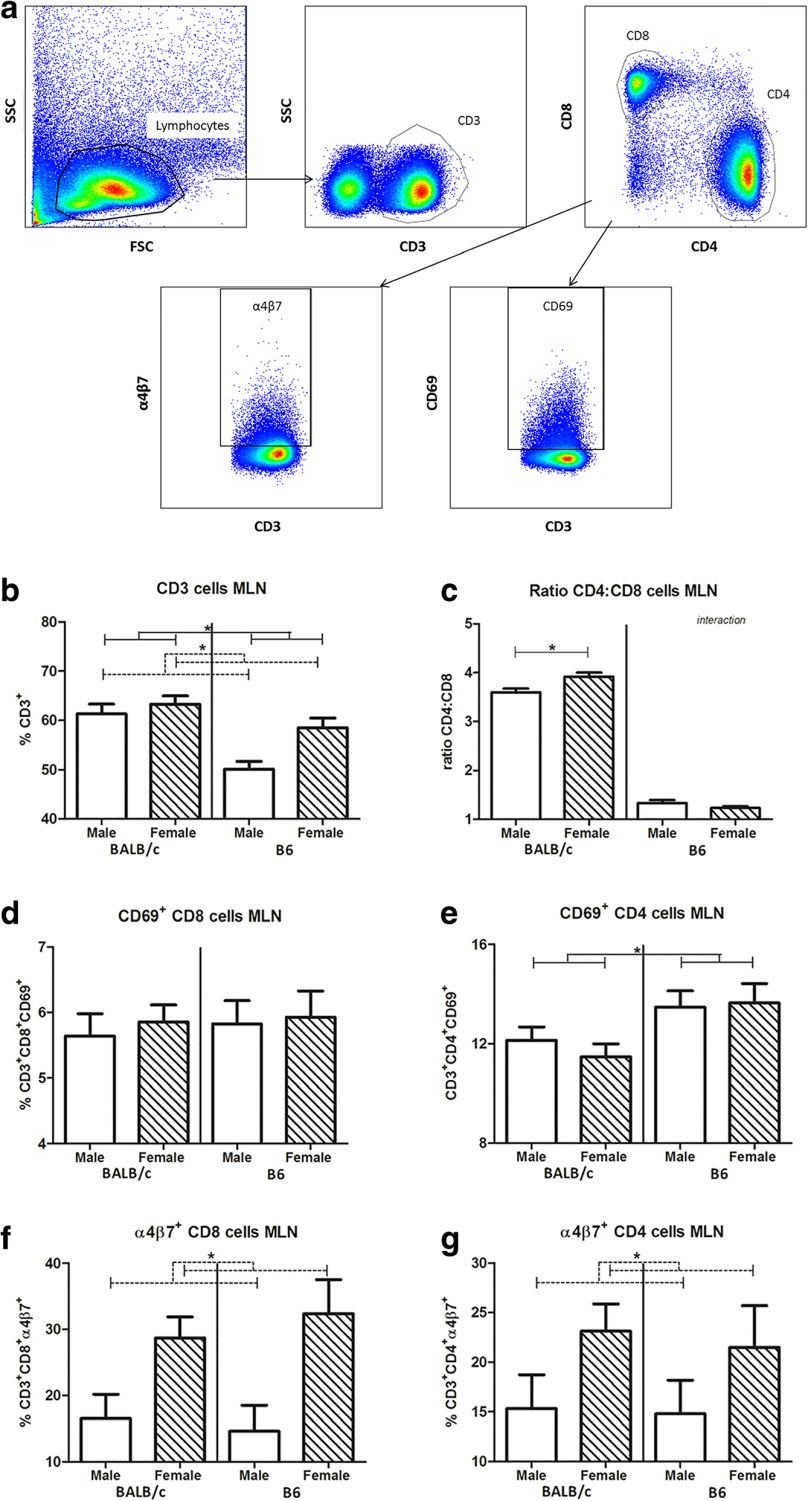
Effect of sex and strain on T lymphocytes in the mesenteric lymph nodes. Gating strategy for determination of T cell subsets in the mesenteric lymph nodes (**a**). Lymphocytes were gated based on size and scatter in the forward side scatter plot. T cells were determined by selecting CD3^+^ cells. Within the CD3^+^ cells, CD8^+^ (Tc cells) and CD4^+^ (Th cells) cells were selected. Within both the CD8^+^ and CD4^+^ population, the percentage of CD69 and α4ß7 were measured. All isotype controls were set at 1%. Frequency of CD3^+^ T lymphocytes (**b**), the ratio of T helper cells (CD4)/T cytotoxic cells (CD8) (**c**), frequency of CD69^+^ CD8 (**d**), CD69^+^ CD4 (**e**), 4β7^+^ CD8 (**f**), and 4β7^+^ CD4 (**g**) in the mesenteric lymph nodes of male and female BALB/c and B6 mice (10 mice per group). T cytotoxic and T helper cells are expressed as the frequency of CD8^+^ and CD4^+^ cells within the CD3^+^ population, respectively. Results are shown as mean + SEM and were tested for overall strain and sex effects using a two-way ANOVA followed by a Bonferroni post hoc test to test for strain-specific sex effects when interaction was found. Significant strain effects are indicated with solid lines and significant sex effects are indicated with dashed lines (*p* < 0.05)

The results of the microarray showed that the expression of several genes which can be related to immunological functions (e.g., T cell trafficking, activation, and maturation) were up- or downregulated by sex (Table 6). Therefore, we evaluated the effect of sex on T lymphocytes in the mesenteric lymph nodes (MLN) using flow cytometry. We chose the MLN, since this is an important site for the induction of intestinal immune responses [32]. We measured the percentages of T lymphocytes, T helper (CD4^+^) cells, and T cytotoxic (CD8^+^) cells. Furthermore, we measured their expression of the early activation marker (CD69), their expression of gut-homing receptor α4ß7 and their maturation status (CD62L and CD44). Overall, male mice had a lower percentage of T lymphocytes in their MLN than female mice (two-way ANOVA, *p* = 0.010) (Fig. 4a). Interaction between sex and strain was found in the CD4^+^/CD8^+^ ratio (two-way ANOVA, *p* = 0.004); BALB/c males had a lower CD4^+^/CD8^+^ ratio than BALB/c females (Bonferroni, *p* < 0.01), while no effect of sex was seen in the B6 strain (Fig. 4b).

**Fig. 5:**
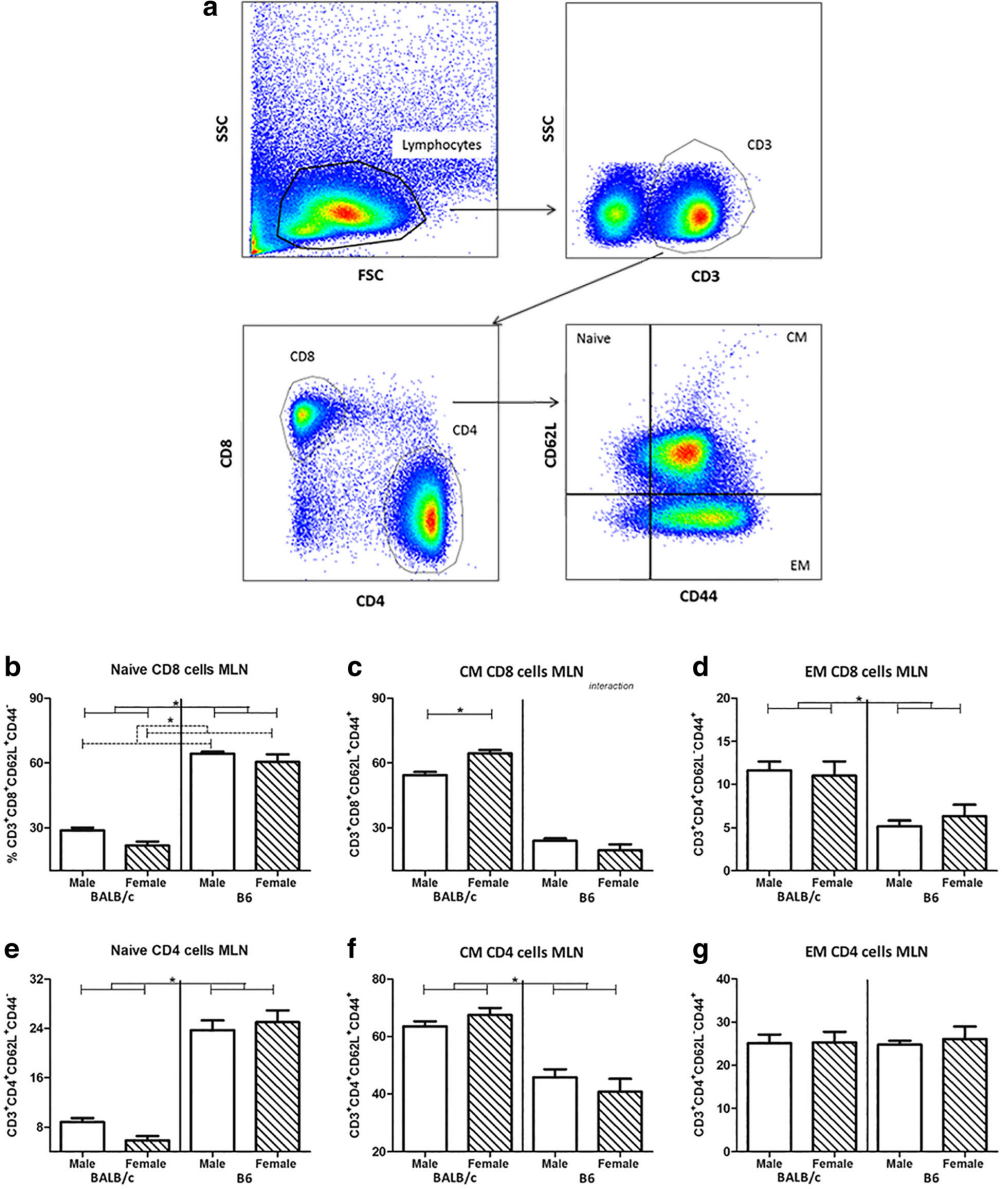
Effect of sex and strain on maturation of T lymphocytes in the mesenteric lymph nodes. Gating strategy for determination of T cell subsets in the mesenteric lymph nodes (**a**). Lymphocytes were gated based on size and scatter in the forward side scatter plot. T cells were determined by selecting CD3^+^ cells. Within the CD3^+^ cells, CD8^+^ (Tc cells) and CD4^+^ (Th cells) cells were selected. Within both the CD8^+^ and CD4^+^ population, the percentage of CD62L and CD44 were measured. All isotype controls were set at 1%. Frequency of CD62L^+^CD44^−^ naive CD8 (**b**) and CD4 (**e**), CD62L^+^CD44^+^ central memory CD8 (**c**) and CD4 (**f**) and CD62L^−^CD44^+^ effector memory CD8 (**d**) and CD4 (**g**) in the mesenteric lymph nodes of male and female BALB/c and B6 mice (10 mice per group). T cytotoxic cells are expressed as the frequency of CD8^+^ cells within the CD3^+^ population, whereas T helper cells are expressed as the frequency of CD4^+^ cells within the CD3^+^ population. Results are shown as mean + SEM and were tested for overall strain and sex effects using a two-way ANOVA followed by a Bonferroni post hoc test to test for strain-specific sex effects when interaction was found. Significant strain effects are indicated with solid lines and significant sex effects are indicated with dashed lines (*p* < 0.05)

The percentage of CD8^+^ or CD4^+^ cells expressing of CD69 was not affected by sex (Fig. 4c, d). The expression of integrin α4ß7 (homing marker) on CD8^+^ and CD4^+^ was lower in male mice as compared to female mice (two-way ANOVA, *p* = 0.001 and *p* = 0.046, respectively) (Fig. 4e, f). In addition, males showed an increased percentage of naïve CD8^+^ cells as compared to females (two-way ANOVA, *p* = 0.031) (Fig. 5a). Interaction was found between sex and strain in the percentage of central memory (CM) CD8^+^ cells (two-way ANOVA, *p* = 0.001); BALB/c males had a lower percentage of CM CD8^+^ cells than BALB/c females (Bonferroni, *p* < 0.01), while sex had no effect on the B6 strain (Fig. 5b). We observed no effect of sex on CD8^+^ effector memory (EM) cells (Fig. 5c). Sex did not influence the percentage of naïve, CM, or EM CD4^+^ cells (Fig. 5d–f).

## Discussion

In this study, we demonstrated clear sex differences in intestinal microbiota, intestinal gene expression, and immune cell composition. We validated the sex effects by using two mouse strains with different genetic backgrounds and microbiota profiles [47]. Although sex significantly explained part of the variance in microbiota composition, this was mouse strain-dependent. Furthermore, we found that the expression of many colonic (mucosal) genes related to immunological functions (e.g., T cell trafficking, activation, and maturation) were up- or downregulated by sex, again in a mouse strain-dependent way. As sex effects in microbiota and sex effects in mucosal gene expression were both strain dependent, we correlated microbiota species with mucosal gene expression data per mouse strain. We found correlations between genes associated with immune populations and certain sex-specific bacteria. Despite these strain-dependent effects of sex on microbiota composition and mucosal immune responses, almost similar sex differences in immune cell populations in the MLN in the two mouse strains were found.

This study demonstrated sex differences in intestinal microbiota composition in healthy mice of two different genetic backgrounds. We showed that sex influenced the microbial diversity and richness and found that males had a lower microbial diversity and richness than females. These findings are in line with our previous study in which we, among others, focused on sex-specific effects of aging on the microbiota composition (B6 mice) [48]. Additionally, this corroborates the findings of Yurkovetskiy et al. and Xiao et al., who also found a higher microbiota diversity in female mice than in male mice [23, 26]. In general, it is assumed that a microbiome with a higher diversity and richness is beneficial for host health [49]. High microbial richness is linked to microbiota stability [49], whereas a lower microbial richness and diversity is linked to several disorders, including obesity [9, 11], and IBD [10]. Therefore, the reduced microbial diversity and richness in male mice in this study may support the results and conclusions of Bábíčková et al. (2015) who found that male mice (B6) have a higher sensitivity to develop DSS-induced colitis (used as IBD model) than female mice (B6) [50]. However, this needs to be confirmed in future studies.

When focusing on the microbiota composition at species-like level we also found sex-specific differences, these were also strain dependent. We found that B6 females had a relative higher abundance of, among others, *Lactobacillus plantarum* and *Bacteroides distasonis et rel.* as compared to B6 males. These species have been shown to influence immune responses, such as enhancing Tregs [51, 52]. BALB/c females had a relative higher abundance of *Bifidobacterium* as compared to BALB/c males. *Bifidobacteria* were shown to have beneficial effects against a variety of gastrointestinal disorders, including colitis [53], and have also been shown to be able to induce regulatory T cells [54]. Also other studies have shown sex-specific microbiota in mice [23, 26–28]. It is interesting to note that the study of Org et al. (2016) also showed that sex differences in the microbiota of mice depends on the genetic background [28]. Pertinent sex differences in microbiota composition in the various studies, however, are difficult to compare, since they are dependent on the strain [28], diet [25] and probably also vendor [55]. Additionally, stress and the gut microbiome also showed to interact which each other and the sensitivity for stress seems to be dependent on genetic background but also sex [56]. Moreover, maternal stress showed to modulate sex differences in the microbiota composition of the offspring [57]. Taken together, stress also may be an interfering factor on dimorphism in microbiota composition and may be either an underling factor or consequence. Our data show that the mouse strain-dependent sex differences in microbiota composition highlight the importance of considering the genetic background when selecting an animal model and the need for standardization on genetic background and other interfering factors in human studies.

The causes of the sex differences in the intestinal microbiota composition are probably multifold. Sex hormones may play a role, since differences in microbiota profiles between males and females in NOD mice disappeared after castration of the males [23], suggesting the involvement of testosterone. However, in view of the strain differences, it is likely that genetic differences also play an important role. Genetic differences might for instance be variations in mucus composition, which affect microbiota composition [58]. Also maternal differences in oligosaccharide composition of mother milk are genetically determined and affect the development of the microbiota composition of the offspring [59, 60]. Whether sex differences in the microbiome also appear in humans is difficult to conclude as human studies on sex differences are still scarce and influenced by many confounding factors. Some human studies found small sex differences in the microbiome [61–64], while others did not [65–67]. Confounding factors might be heterogeneity in genetics, but also the reproductive condition of females (e.g., menstrual cycle, the use of oral contraceptives, and menopause), which is often not taken into account. Such factors can not only interfere with immune responses [68, 69], and with microbiota composition, but may also modulate the sex effects.

A major goal of this study was to correlate sex-specific intestinal immune differences with specific microbiota. The bacteria and intestinal genes that were selected for this correlation were the most explanatory for variation between sex and mouse strain. For B6 mice, we showed that various female-specific bacteria positively correlated with one cluster of genes, which were, among others, associated with inflammatory responses and leukocyte migration. In BALB/c mice, we found four clusters of genes correlating with various male specific bacteria (bacteria which were or tended to be significantly increased in males), which were involved in, among others, differentiation of lymphocytes and expansion of helper T cells, while the clusters which were negatively correlating with the male bacteria were involved in, among others, chemotaxis and quantity of phagocytes. Our data do suggest that microbiota may influence immunological gene expression in the gut. Although genes in the pathway of expansion of T helper cells were positively correlated with various bacteria increased in BALB/c males, this does not necessarily mean that this is associated with increased numbers of T helper cells, since genes in this pathway may also inhibit expansion of T helper cells. Indeed, our flow cytometry data show decreased numbers of T helper cells in the MLN in BALB/c mice.

Our study was merely observational. However, another study from our lab showed that sex-specific microbiota may indeed affect immune responses. Fransen et al. (2017) performed a microbiota transfer study in germ-free mice by transferring male microbiota into female germ-free mice and female microbiota into germ-free male mice [70]. Fransen observed that germ-free male recipients of male microbiota had a higher percentages of RORγt^+^ Foxp3^+^ cells in the PPs and MLN as compared with germ-free male recipients of female microbiota, indicating that indeed sex differences in microbiota may induce sex differences in immune responses [70]. However, they also found that males in general had a higher percentages of conventional Tregs, independent of whether they received microbiota from male or female mice, suggesting not all sex differences in immune response are dependent on the microbiome [70].

We also found sex differences in immune cell populations in the MLN of both strains and despite the different sex effects in the microbiota and gene expression in the two strains, we observed similar sex differences in immune cell populations in the MLN in both strains, and also in the spleen (21). Analysis of the microarray data showed that the expression of several genes which can be related to immunological functions (e.g., T cell trafficking, activation, and maturation) were up- or downregulated by sex. Therefore, we focused on T cells and T cell functions in the MLN, and we found that females had a higher percentage of total T cells, with an increased percentage of these T cells expressing the homing receptor α4β7 (T cell trafficking) than males. Furthermore, female mice had a lower percentage of naive T cytotoxic cells (T cell maturation) than male mice. The higher percentage of T cells and lower percentage of naïve T cells fits with the general idea that females have a stronger adaptive immune arm than males [22]. It is unknown from this study how these changes are induced, but as indicated above, sex differences in microbiota species may be involved. Also in the MLN, we found that some of the sex effects in the immune cell composition were strain dependent, although less apparent: only the CD4^+^/CD8^+^ ratio and central memory CD8^+^ cells were affected by sex in a strain-dependent way. Similar results were recently found in the Peyer’s patches (PP) and the spleen: sex effects on immune cell populations were mainly strain independent [21].

## Conclusions

This study demonstrated sex differences in intestinal microbiota species, diversity, and richness in healthy mice of two different mouse strains. The nature of the sex effects, however, appeared to be determined by the mouse strain, since different bacterial species were enriched in males and females in the two strains. The strain-dependent sex effects were also observed in the expression of immunological genes in the colon. The correlations we found between male and female specific bacteria with various immunological gene pathways, suggest that sex differences in the microbiome may be involved in sex differences in immune responses. To our opinion, this is an important observation and not only implies that preventive measures for disease development may require a sex- and genetic-specific approach, but it also shows that in microbiome studies, both sex and genetic background should be taken into consideration. Our study may also shed light on the conflicting results in studies with respect to sex differences in the microbiome, especially in human studies. Conflicting results found in human studies [25, 61–67] may be due to the lack of standardization with respect to sex and genetic background.

## Additional file

Additional file 1: Method and results RT qPCR for validation microarray. (DOCX 27 kb)

## Abbreviations

B6: C57B1/6OlaHsd mice
BALB/c: Balb/cOlaHsd mice
CM: Central memory cells
DSS: Dextran sulfate sodium
EM: Effector memory cells
FCS: Fetal calf serum
IBD: Inflammatory bowel disease
IBMT: Intensity-based moderated *t* statistic
IPA: Ingenuity pathway analysis
MITChip: Mouse intestinal tract chip
MLN: Mesenteric lymph nodes
NOD: Non-obese diabetic
PLS: Partial least squares
PP: Peyer’s patches
RBB + C: Bead-beating-plus column
RDA: Redundancy analysis
RPA: Robust probabilistic averaging
SCFAs: Short-chain fatty acids
Th: T helper cells
Tregs: T regulatory cells
