# Supplementary material for: Sex and strain dependent differences in mucosal immunology and microbiota composition in mice
Source: Biol Sex Differ. 2018 Jun 18;9:26. doi: 10.1186/s13293-018-0186-6 (PMC6006852; doi:10.1186/s13293-018-0186-6)
Supplement: Supplementary file 1 — Method and results RT qPCR for validation microarray. (DOCX 27 kb) [file 13293_2018_186_MOESM1_ESM.docx]

**Additional file 1**

1. **Methods**
   1. **RT qPCR**

A real-time quantitative PCR (RT qPCR) was performed to validate the microarray data. RNA was purified from the proximal colon of mice (n=5 per group) using TRIzol (Life Technologies, Calsbad, CA, USA) followed by an additional round of purification with RNeasy Minikit columns (Qiagen, Venlo, the Netherlands). The quality of RNA was determined using RNA 6000 nanochips on the Agilent 2100 bioanalyzer (Agilent Technologies, Amsterdam, the Netherlands). RNA concentrations were determined using a NanoDrop 1000 spectrophotometer (NanoDrop products, Wilmington, USA). RNA (500 ng) was reverse-transcribed to cDNA using a SuperScript® II Reverse Transcriptase kit according to the instructions of the manufacturer (Life technologies). cDNA was stored at -20 °C until further use. Gene expression levels of 12 genes were evaluated with RT qPCR (see table S1). These included the top five immunological genes in each mouse strain which differed significantly between the males and females in the microarray. Besides, we validated one gene per strain which had the most significantly different expression between males and females (this was a gene on the Y or X chromosome) (as a positive control) and one gene which had no significantly different expression between males and females (as a negative control).

**Table S1.** Selected of immunological genes for validation gene array with RT qPCR.

|  | **Selection criteria** | **Gene ID** |
| --- | --- | --- |
| **Genes B6** |  |  |
| *Apoe* | Selection from immune genes | 11816 |
| *Cd3e* | Selection from immune genes | 12501 |
| *Bach2* | Selection from immune genes | 12014 |
| *Ccr2* | Selection from immune genes | 12772 |
| *Cd74* | Selection from immune genes | 16149 |
| *Uty* | Positive control | 22290 |
| *Ccr6* | Negative control | 12458 |
| **Genes BalbC** |  |  |
| *Batf* | Selection from immune genes | 53314 |
| *Ccr9* | Selection from immune genes | 12769 |
| *Ccr2* | Selection from immune genes | 12772 |
| *Ccr6* | Selection from immune genes | 12458 |
| *Cd86* | Selection from immune genes | 12524 |
| *Xist* | Positive control | 213742 |
| *Cd226* | Negative control | 225825 |

RT qPCR was conducted in triplicates using ViiA7 Real-Time PCR system (Thermo Fisher Scientific) using the following program: 2 min 50 °C and 10 min 95 °C, followed by 40 cycles: 15 sec. 95 °C and 1 min 60 °C. Primers/probe sets (Taqman Gene expression) were obtained from Thermo Fisher Scientific) (Table S2) and the analysis was performed in triplicates. The difference in relative gene expression (2^-(ΔCt)) between males and females within both strains were determined with a t-test (p<0.05). The housekeeping gene *Ppib* was used as an internal control [1].

**Table S2**. Primers for RT qPCR.

| **Gene symbol** | **Assay ID** |
| --- | --- |
| *Cd3e* | Mm01179194_m1 |
| *Apoe* | Mm01307193_g1 |
| *Ccr2* | Mm99999051_gH |
| *Bach2* | Mm00464379_m1 |
| *Cd74* | Mm00658576_m1 |
| *Ccr6* | Mm99999114_s1 |
| *Uty* | Mm00447710_m1 |
| *Cd86* | Mm00444540_m1 |
| *Batf* | Mm00479410_m1 |
| *Ccr9* | Mm02528165_s1 |
| *Cd226* | Mm01301769_m1 |
| *Xist* | Mm01232884_m1 |
| *Ppib* | [Mm00478295_m1](https://www.thermofisher.com/taqman-gene-expression/product/Mm00478295_m1?CID=&ICID=&subtype=) |

1. **Results**
   1. **Microarray validation**

We performed a real-time quantitative PCR (RT qPCR) to validate the microarray data. For the B6 mice, the RT qPCR showed similar fold changes between males and females for all five selected immunological genes as the microarray (Table S3). For the Balb/c mice, the RT qPCR showed similar fold changes between males and females for three of the five selected immunological genes as the microarray (Table S4). The other two genes (*Batf* and *Ccr6*) did not have a significant different relative expression between the males and the females.

**Table S3.** Relative expression of genes in male and female B6 mice determined with a gene array and qPCR. The difference in relative gene expression between the males and females within both strains were determined with a t-test.

|  | **Relative expression gene array** | | | **Relative expression qPCR** | | |
| --- | --- | --- | --- | --- | --- | --- |
| **Gene symbol** | **B6 female** | **B6 male** | **t-test** | **B6 female** | **B6 male** | **t-test** |
| *Apoe* | 3188.8 | 2024.1 | p<0.05 | 2.0360 | 1.2150 | p<0.05 |
| *Cd3E* | 110.7 | 74.4 | p<0.05 | 0.0187 | 0.0108 | p<0.05 |
| *Bach2* | 66.6 | 38.5 | p<0.05 | 0.0174 | 0.0077 | p<0.05 |
| *Ccr2* | 55.5 | 39.0 | p<0.05 | 0.0696 | 0.0395 | p<0.05 |
| *Cd74* | 1520.0 | 918.0 | p<0.05 | 2.6510 | 1.6150 | p<0.05 |
| *Uty* | 7.3 | 257.7 | p<0.05 | ND | 0.0853 |  |
| *Ccr6* | 19.3 | 18.6 |  | 0.0018 | 0.0009 |  |

ND= not detectible

**Table S4.** Relative expression of genes in male and female B6 mice determined with a gene array and qPCR. The difference in relative gene expression between the males and females within both strains were determined with a t-test.

|  | **Relative expression gene array** | | | **Relative expression qPCR** | | |
| --- | --- | --- | --- | --- | --- | --- |
| **Gene symbol** | **BalbC female** | **BalbC male** | **t-test** | **BalbC female** | **BalbC male** | **t-test** |
| *Batf* | 56.9 | 70.3 | p<0.05 | 0.0057 | 0.0083 |  |
| *Ccr9* | 34.8 | 45.1 | p<0.05 | 0.0254 | 0.0295 | p<0.05 |
| *Ccr2* | 56.8 | 84.6 | p<0.05 | 0.0639 | 0.0868 | p<0.05 |
| *CR6cr* | 23.4 | 35.1 | p<0.05 | 0.0020 | 0.0061 |  |
| *Cd86* | 66.8 | 88.7 | p<0.05 | 0.0159 | 0.0247 | p<0.05 |
| *Xist* | 4322.4 | 28.9 | p<0.05 | 2.7740 | 0.0009 | p<0.05 |
| *Cd226* | 33.6 | 33.6 |  | 0.0032 | 0.0042 |  |

**References**

1. Sirakov M, Borra M, Cambuli FM, Plateroti M. Defining suitable reference genes for RT-qPCR analysis on intestinal epithelial cells. Mol Biotechnol. 2013;54 3 :930-8; doi: 10.1007/s12033-012-9643-3 [doi].
